# Editorial: Advances in wound repair and regeneration: novel materials, targets and applications

**DOI:** 10.3389/fchem.2024.1487091

**Published:** 2024-09-19

**Authors:** Xinwei Cheng, Jia Zhou

**Affiliations:** Department of Plastic and Reconstructive Surgery, Shanghai Ninth People’s Hospital, Shanghai Jiao Tong University School of Medicine, Shanghai, China

**Keywords:** wound healing, wound regeneration, novel materials, transformation, stem cell, scarring

In the realm of healthcare, chronic wounds and pathological scarring represent a formidable challenge that not only mars aesthetics but also significantly impacts motor function, profoundly affecting patients’ quality of life. From diabetic ulcers and pressure sores to scars from trauma or surgery, these conditions impose a heavy burden on healthcare systems globally (McDermott et al., 2023; Šuca et al., 2024; Lindholm and Searle, 2016). Innovative solutions that accelerate wound healing, minimize scarring and improve patient outcomes are urgently needed. Wound repair and regeneration is an important frontier, and the convergence of materials science, biotechnology and clinical medicine is the key to what promises to be a change in therapeutic wound healing strategies. And the Research Topic “Advances in Wound Repair and Regeneration: Novel Materials, Targets and Applications” aims to be at the forefront of this transformative journey.

This Research Topic aims to bring together a comprehensive Research Topic of the latest advances in the field of wound repair and regeneration, emphasizing the key role of novel materials, therapeutic targets, and innovative applications. By focusing on cutting-edge discoveries and groundbreaking research findings in the field, we hope to foster interdisciplinary collaborations, open up new avenues of research, and accelerate the translation of laboratory results into clinical practice.

One of the most promising materials in wound healing research are hydrogels, which mimic the native extracellular matrix, providing a supportive ecological niche for stem cell survival, proliferation, and differentiation, enhancing the therapeutic potential of stem cells in chronic wound repair (Li et al., 2024). The review by Li et al. focuses on the emergence of hydrogel scaffolds as a multifunctional platform for stem cell delivery and wound microenvironmental modulation. This article reviews the significant strides made in the development of hydrogel dressings tailored for stem cell therapies and demonstrates the great potential of hydrogels combined with stem cells in chronic wound healing. As stem cell and regenerative medicine research deepens, these hydrogel dressings for wound repair are an important area for future investigations.

Another notable contribution comes from the study by Zhang et al., which comparatively analyzed the therapeutic effects of two promising autologous extracts, cell-free fat extract (CFE) and platelet-rich plasma (PRP), on wound healing. By systematically evaluating the mechanism of action, efficacy, and safety of these two autologous, this study provides valuable insights into the relative merits of these two therapies. This comparison is critical to guide clinical decision-making and advances personalized medicine strategies in wound repair.

Diabetic ulcers are a particularly vexing challenge in chronic wound healing. The study by Liu et al. describes a novel graphene oxide hydrogel that combines two distinct cross-linked networks, capable of releasing glutathione in response to fluctuations in glucose concentration and/or reactive oxygen species (ROS) levels. This innovative material effectively mitigates oxidative stress and regulates glucose levels, presenting great potential for the treatment of diabetic ulcers.

Pathological scarring is a common result of poor wound healing, and current treatments continue to face significant challenges such as low efficacy, high side effects, and a propensity for recurrence. The review by Liu et al. focuses on the use of two emerging technologies [microneedling (MN) and photodynamic therapy (PDT)] alongside two novel materials [photosensitizers and exosomes (Exos)] for the treatment of pathological scarring. The new techniques and materials hold promise in enhancing treatment efficacy, mitigating side effects associated with conventional therapies, and reducing the recurrence rate, thereby improving patient outcomes.

Collectively, the articles in this Research Topic offer a vibrant portrayal of the current landscape of wound repair and regeneration research. They demonstrate the background and substantial progress made in this field, fueled by novel materials, innovative therapies, and cutting-edge technologies. By fostering interdisciplinary discourse and encouraging the exploration of new therapeutic avenues, we hope that this Research Topic will provide impetus for new therapeutic ideas and their eventual clinical translation. As the frontiers of wound repair and regeneration continue to expand, the future holds great promise for improving the lives of patients suffering from chronic wounds and pathological scarring.
